# Seasonal and multi-functional use of industrial space at Iron Age Tel Shiqmona, Israel

**DOI:** 10.1371/journal.pone.0355104

**Published:** 2026-09-24

**Authors:** Isaac Ogloblin Ramirez, Golan Shalvi, Sonia Pinsky, Madeline Jacobson, Roni Zuckerman-Cooper, Ayelet Gilboa, David E. Friesem

**Affiliations:** 1 Interdisciplinary Centre for Archaeology and the Evolution of Human Behaviour, Universidade do Algarve, Faro, Portugal; 2 Department of Anthropology, Anthropology Laboratory of MicroArchaeology (ALMA), Rutgers, The State University of New Jersey, New Jersey, United States of America; 3 The Leon Recanati Institute for Maritime Studies, Department of Maritime Civilizations, School of Archaeology and Maritime Cultures, Faculty of Humanities, University of Haifa, Israel; 4 Zinman Institute of Archaeology, School of Archaeology and Maritime Cultures, Faculty of Humanities, University of Haifa, Haifa, Israel; 5 Department of Archaeology, University of Cambridge, United Kingdom; 6 Haifa Center for Mediterranean History, University of Haifa, Israel; Israel Antiquities Authority, ISRAEL

## Abstract

Iron Age industrial complexes in the Southern Levant are frequently perceived as static, mono-functional spaces. This study challenges such perceptions by reconstructing the dynamic life history of the 8^th^-century BCE level of the industrial compound at Tel Shiqmona, the largest known Iron Age purple-dye factory in the Mediterranean and a state-controlled economic asset of the Kingdom of Israel. We conducted a high-resolution investigation of the site’s industrial complexity using micro-geoarchaeological methods, including FTIR spectroscopy, sediment micromorphology, and quantification of micro-remains. The results demonstrate that these spaces were used for a diverse, non-simultaneous range of activities, and they support a model in which this use was structured by the seasonal cycle: olive pressing in late autumn, livestock sheltering in winter, and textile production in spring. Purple-dye production was most probably integrated into this cycle during complementary temporal windows. Additionally, the sequences show evidence of periodic floor management, where wood ash was used as a neutralizing agent to maintain the industrial space between seasonal shifts. We argue that this documented multi-functionality was a manifestation of the sophisticated management and optimization of the kingdom’s economic assets. Ultimately, these findings reveal that the economy of the Kingdom of Israel relied on advanced space-time management, where high-prestige industries and agricultural needs were interwoven into a singular, highly efficient economic machine. More broadly, these findings highlight a systematic interpretive distortion: the archaeological characterization of industrial spaces tends to privilege their most visible activity, even when that activity was constrained to a narrow seasonal window. The operational logic of ancient economic spaces — how they were managed, repurposed, and sustained across the full annual cycle — is recoverable only through high-resolution archaeological analysis in which micro-geoarchaeological methods provide a critical additional layer of resolution that conventional macroscopic analysis alone cannot supply.

## Introduction

### Industrialization during the Iron Age

The Iron Age II in the Southern Levant was characterized by the consolidation of territorial kingdoms and the emergence of complex economic systems (e.g., [1–8]). A hallmark of this period was the development of specialized, large-scale production centers for commodities such as olive oil, wine, and textiles, alongside metal-working industries. While traditional household production continued, the appearance of dedicated industrial zones marks a significant shift towards centralized or elite-controlled economies [4,5,9–16]. Scholarly discussion has often focused on defining the scale of this “industrialization” and identifying the architectural and artifactual footprints of these workshops [17–22].

Such large-scale, specialized production did not emerge in a vacuum but belongs to a wider landscape of intensified commodity production across the Iron Age southern Levant. In the arid southeast, copper was produced at an industrial scale in the Faynan region, including Khirbat en-Nahas, and in the Timna Valley, mobilizing substantial labor and systematic fuel-procurement regimes [22, 23]. This copper industry, however, largely predates the broader expansion of such production in the Iron Age II [24], and the political framework within which it operated remains contested [e.g., 25]. Closer and broadly contemporaneous parallels, embedded within settled communities, are found further north: at Tel Rehov an apiary operating on an industrial scale was installed within the urban fabric, attesting to the organized production of honey and beeswax already in the Iron Age IIA [26], and iron workshops of comparable date are attested in Philistia (Tell es-Safi/Gath; [27,28]) and in Transjordan, where the substantial smelting operation at Tell Hammeh was active around 900 BCE [29]. Set against this regional landscape of intensified industry, the purple-dye industry of Tel Shiqmona represents another expression of the same broader phenomenon, though here the principal driver was, as we argue below, the growing Mediterranean demand for purple dye.

The archaeological record often captures a specific moment in time, usually associated with a site’s final state of destruction or abandonment, and tends to privilege its most archaeologically visible activities over less visible uses of space. While contemporary archaeology widely recognizes that floor assemblages are not frozen snapshots of past activity but complex products of cultural and natural formation processes, including curation, abandonment behavior, reuse, and post-depositional mixing [30,31] (cf. the ‘Pompeii Premise’ [32]), this awareness has not consistently reshaped how industrial spaces are interpreted in practice. When evidence for a seasonal industry such as olive-oil pressing is recovered, the question of how the same space was used during the greater part of the year, when that industry stood idle, is rarely placed at the center of the interpretive effort, even though it is precisely that off-season use that accounts for most of the space’s working life. The resulting reconstructions tend to portray industrial (and other) spaces as static and mono-functional, overlooking the dynamic rhythms of daily life that likely fostered a diversity of activities. The few studies that have considered complementary, off-season use of such spaces remain exceptions (e.g., [18, 33,34:n.1]). Relatedly, the impact of seasonality remains a largely neglected dimension in archaeology [35:5], leading to reconstructions of ancient production that overlook the seasonal cycles and fluctuating material availability that governed the use of space. Since the macroscopic artifactual record often lacks the stratigraphic resolution necessary to detect intermittent use or secondary activities—such as short-term provisional animal penning or temporary storage—our understanding of how industrial complexes operated on a seasonal basis remains fragmentary. Some recent work has begun to address the functional complexity of Iron Age spaces more systematically (e.g., [36]), yet the seasonal dimension remains largely unexplored.

In recent years, advanced high-resolution methodologies have been increasingly integrated into archaeological research to identify complex micro-processes invisible to the naked eye (e.g.[37–41]). However, while seasonality is a core investigative pillar in prehistoric scholarship (e.g., [41–46]), Bronze and Iron Ages research in the Levant has seen only limited attempts to address this temporal dimension empirically, (but see [47], albeit from the Byzantine period) and the issue of seasonality in these periods has remained a largely theoretical hypothesis. Beyond these isolated hints, empirical studies have focused on environmental extremes: either the opportunistic mobility of semi-nomadic societies in arid regions, driven by seasonal precipitation patterns (e.g.[23,48]), or the biological constraints of crops like olives [15]. Similarly, where seasonality has been addressed in sedentary societies beyond the Southern Levant—whether through residential organization in Scandinavian longhouses (though from a later Iron Age context [49]) or transhumance patterns in Western Europe (see various contributions in [50,51])—the focus has remained on pastoral mobility rather than on the multi-functional scheduling of permanent industrial spaces. Even in unique industrial contexts like the Hallstatt salt mines in Austria, seasonal insights focused on the seasonal diet of laborers rather than the internal spatial organization and functional rotation of the production site [52]. In these contexts, seasonality is typically viewed as a response to environmental triggers, such as winter rainfall, pastoral cycles, or crop ripening.

Consequently, the seasonal operational logic of permanent settlements and the ways in which industrial spaces were optimized throughout the annual cycle remain largely unexplored. We present a study of the fortified industrial outpost at Tel Shiqmona, a site primarily dedicated to the prestigious purple-dye industry. By employing micro-geoarchaeological techniques—including sediment micromorphology and the analysis of micro-remains (phytoliths, wood ash, and dung residues)— this study examines human activities and their depositional contexts within Shiqmona’s industrial compound. Integrated with archaeological and historical data, the micro-geoarchaeological evidence is used to interpret the use of space dividing the industrial area into task-specific zones, and discussing their possible scheduling and dynamic history, contributing to a broader understanding of the seasonal rhythms and economic organization of production at the site. Two related but distinct concepts frame this study: multi-functionality, the use of a single architectural space for qualitatively different activities; and seasonality, the organization of these activities according to the annual cycle. The first is directly attested by macro- and micro-remains; the second is an inference from the known annual timing of each activity.

### Tel Shiqmona: Archaeological and historical background

**Fig 1:**
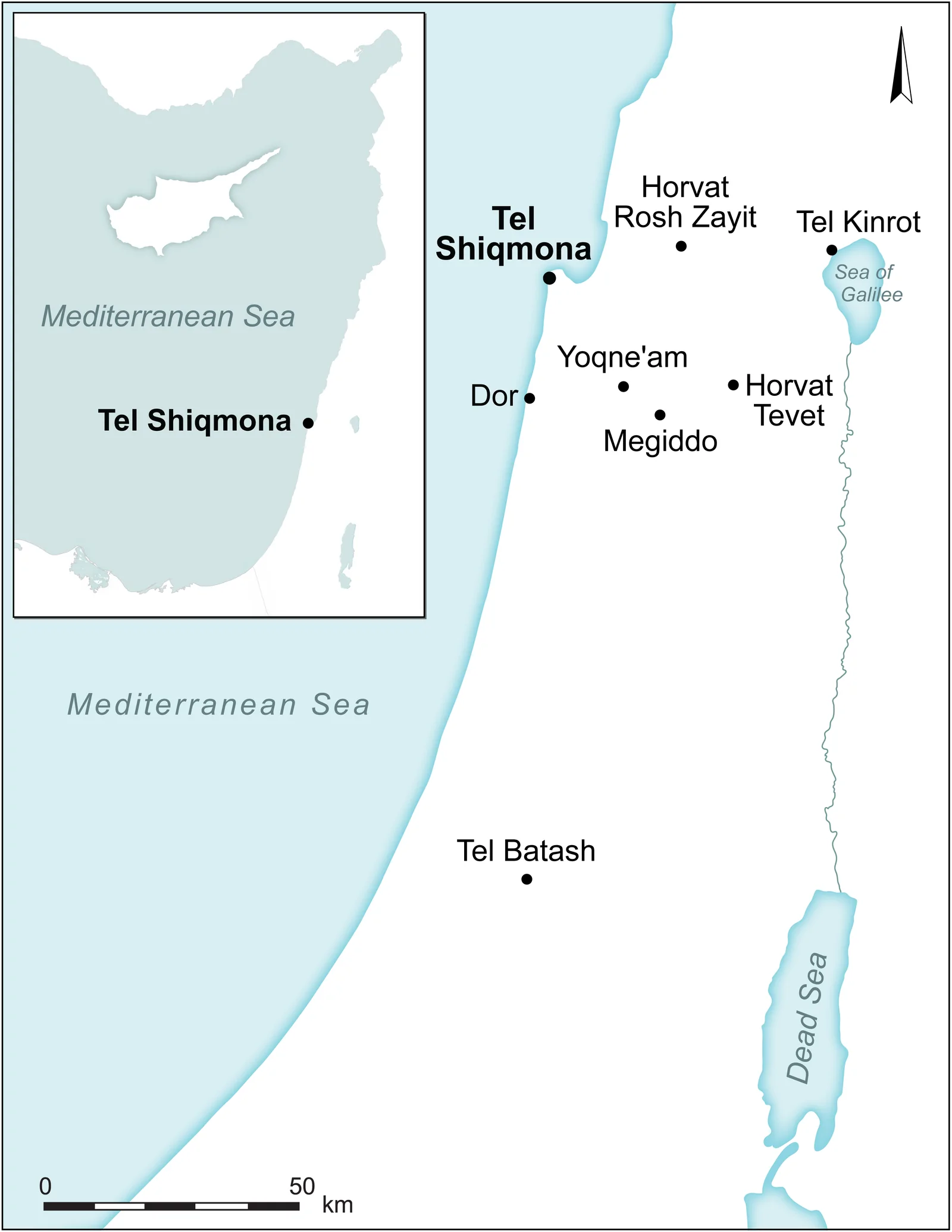
Location map of Tel Shiqmona and the other sites mentioned in the text; inset, the eastern Mediterranean basin. The map is an original schematic drawing prepared for this study and contains no satellite imagery or proprietary base-map data.

Tel Shiqmona (Arabic: Tell es-Samak) located on the Carmel coast of Israel (Fig 1), is unique in the Iron Age archaeological landscape of the Mediterranean basin. It is a very small site, approximately two acres large, functioning as a specialized, large-scale production center, the largest known Iron Age purple-dye factory in the Mediterranean [53–55]. Its geographical location on a rocky promontory was decisive; while the rocky reef hindered its potential as an anchorage, it offered the optimal ecological niche for the *Muricidae* snails —the source of Mediterranean purple dye—facilitating the establishment of this high-volume, prestigious industry that operated continuously for centuries [53].

The site was excavated by Joseph Elgavish in the 1960s and 1970s, by Shay Bar for three seasons (2011–2013), and most recently by Golan Shalvi in 2023. Excavations have revealed a well-preserved complex for purple-dye production and fiber-dyeing that also contains evidence of additional crafts, primarily olive oil production and textile weaving [56–58], raising questions about the spatial and temporal organization of labor.

The identification of multi-functional and seasonal space utilization at Shiqmona is not merely a reconstruction of local activities, but is essential to understanding the site’s strategic role within the broader economic landscape of the Kingdom of Israel and its integration into the wider Mediterranean trade network. During the late Iron Age IIA and IIB (Strata 13–11; ca. 870/850–740 BCE), Shiqmona appears to have functioned as a specialized economic asset of the Kingdom [55], not unlike other royal estates such as Horvat Tevet [16] and Horvat Rosh Zayit [59]. The ability to operate multiple industries within a single, relatively small building complex suggests sophisticated optimization of labor, space, and other resources. In the specific context of Shiqmona, this spatial elasticity was not merely an option but a logistical necessity; given the site’s extremely confined area (approx. two acres), every architectural unit had to be optimized to its full potential throughout the annual cycle.

### Stratum 11: The peak of industrial activity

**Fig 2:**
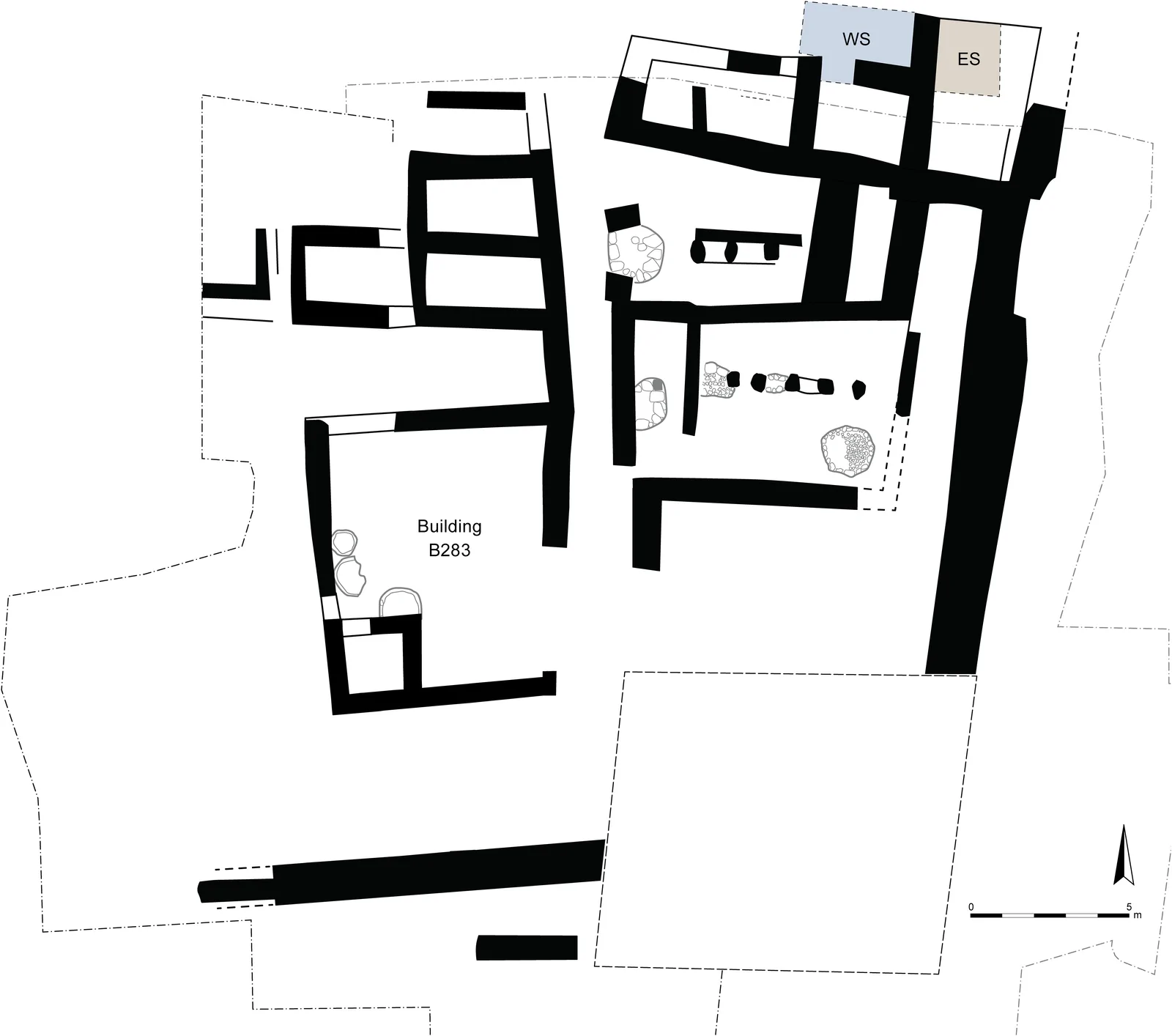
Schematic plan of Tel Shiqmona Stratum 11, incorporating the Elgavish, Bar, and Shalvi excavations. The areas where the micro-geoarchaeological study was conducted are demarcated in color at the top of the plan (2023 excavation). Illustration by Sapir Haad.

This study focuses on the 2023 excavation season in which Stratum 11 deposits were excavated (Fig 2). In the Tel Shiqmona sequence, Stratum 11 represents the zenith of Iron Age activity. This stratum spanned approximately 50 years (ca. 790–740 BCE), corresponding with the reign of Jeroboam II in the Kingdom of Israel.

The material record of Stratum 11 is exceptionally rich, yielding over 40 artifacts related to purple-dye production, with more than half of them stained with the dye itself, providing direct evidence of the industry. These comprise evidence for at least 16 specialized dyeing vats, each with a capacity of approximately 350 liters (though not necessarily in simultaneous use [53]). Additionally, at least two olive-oil presses are securely attributed to this stratum, with a third potentially associated with it [57: Supplementary Material 2]. The occupation ended in violent destruction, leaving several assemblages in primary deposition.

**Fig 3:**
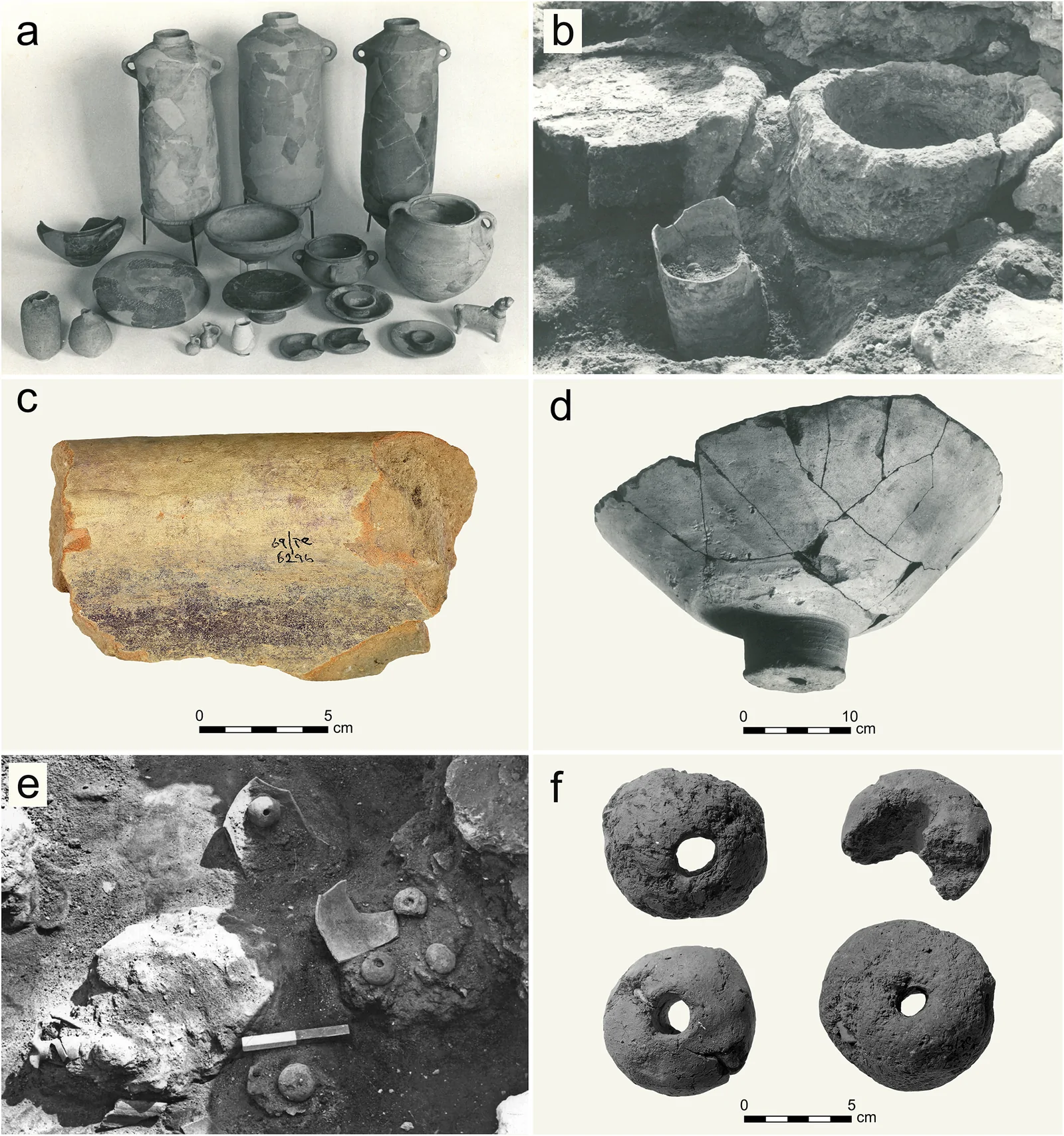
Finds from Building B283, Stratum 11, Tel Shiqmona. (a) The complete vessel assemblage recovered from the building. (b) Olive-press installation *in situ*, comprising a crushing mortar, a stone press-bed, and a ceramic collecting jar (basket 6391). (c) Vat rim sherd with purple-dye staining (basket 6296). (d) Base of the largest dye vat recovered in the excavation (basket 6296). (e) Loom weights *in situ* (basket 6224). (f) Additional selected loom weights (basket 6238).

Building B283, exposed in Elgavish’s excavations (Fig 2), is a representative and exceptionally well-preserved architectural unit of Stratum 11 that yielded a rich ceramic assemblage (Fig 3:a) and in which the site’s principal industries—purple dye, olive oil, and textiles—are represented within a single space [57]. It yielded an olive-oil press (Fig 3:b) [57: Supplementary Material 2, p. 8] and abundant evidence of a purple-dye industry, including a rim bearing purple-dye residue, large restored lower part of a purple-dye vat, and other potsherds and stones that were similarly stained (see, for example, the base and rim in Fig 3:c-d; [53]), together with a cluster of sixteen uniform clay loom weights recovered *in situ* (Fig 3:e-f), best understood as the remains of one or two warp-weighted looms [58]. Olive-oil pressing, purple-dye production, and textile manufacture are thus documented side by side within the same destruction layer of a single architectural unit. The macroscopic coexistence of the three industries within Stratum 11 is therefore beyond doubt; what those earlier, coarsely excavated contexts could not resolve was the fine-grained history of how the space was used and maintained over time. The high-resolution, micro-geoarchaeological excavation described below was designed to recover precisely this, within a single concentrated area.

## Materials and methods

### Fieldwork and macro-finds

**Fig 4:**
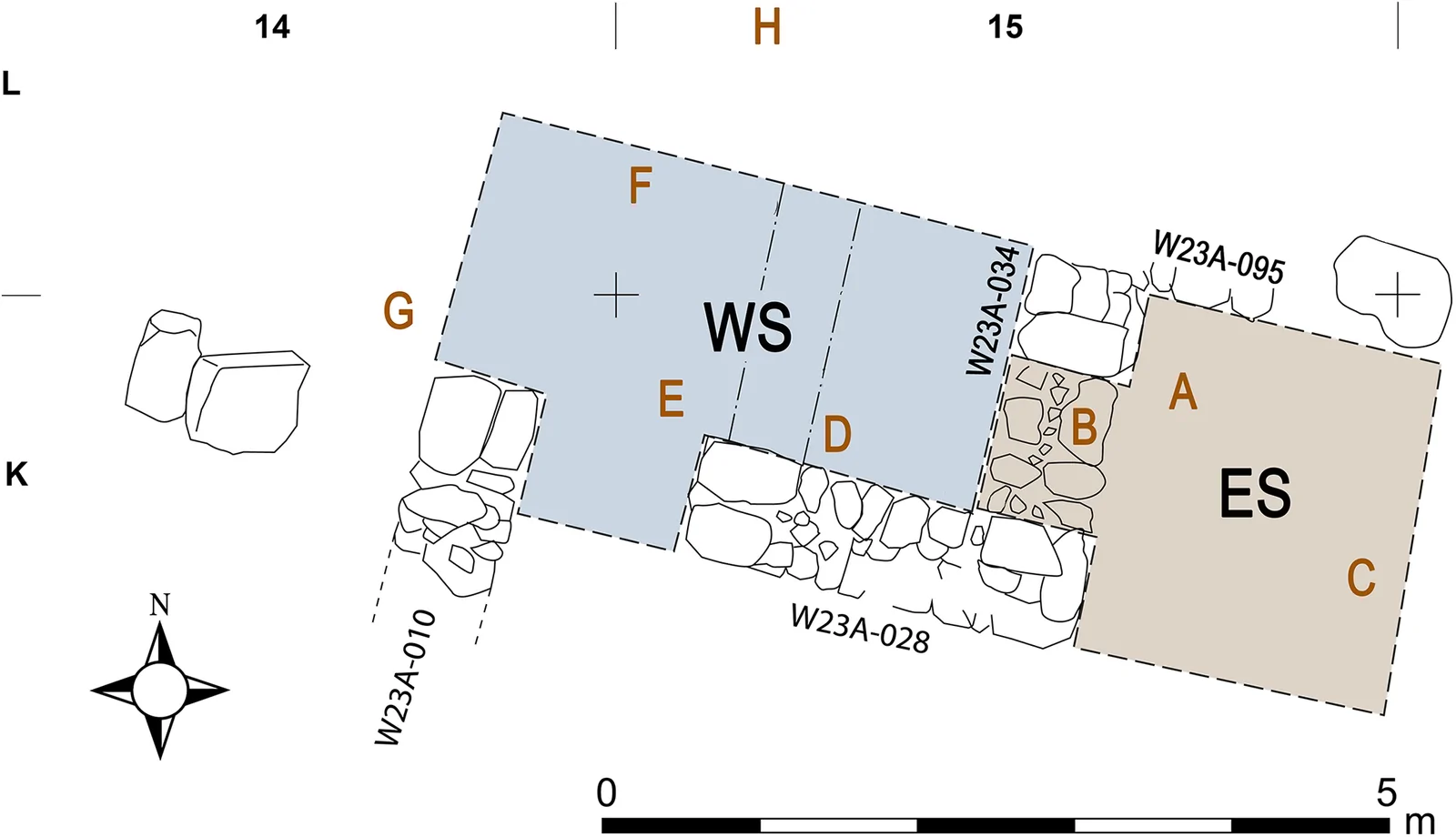
The top plan shows the Eastern Space (ES) and Western Space (WS). The letters A–H indicate the findspots of the artifacts in the order of their appearance in Fig 5. Plan by Sveta Matskevich; graphic processing by Sapir Haad.

**Fig 5:**
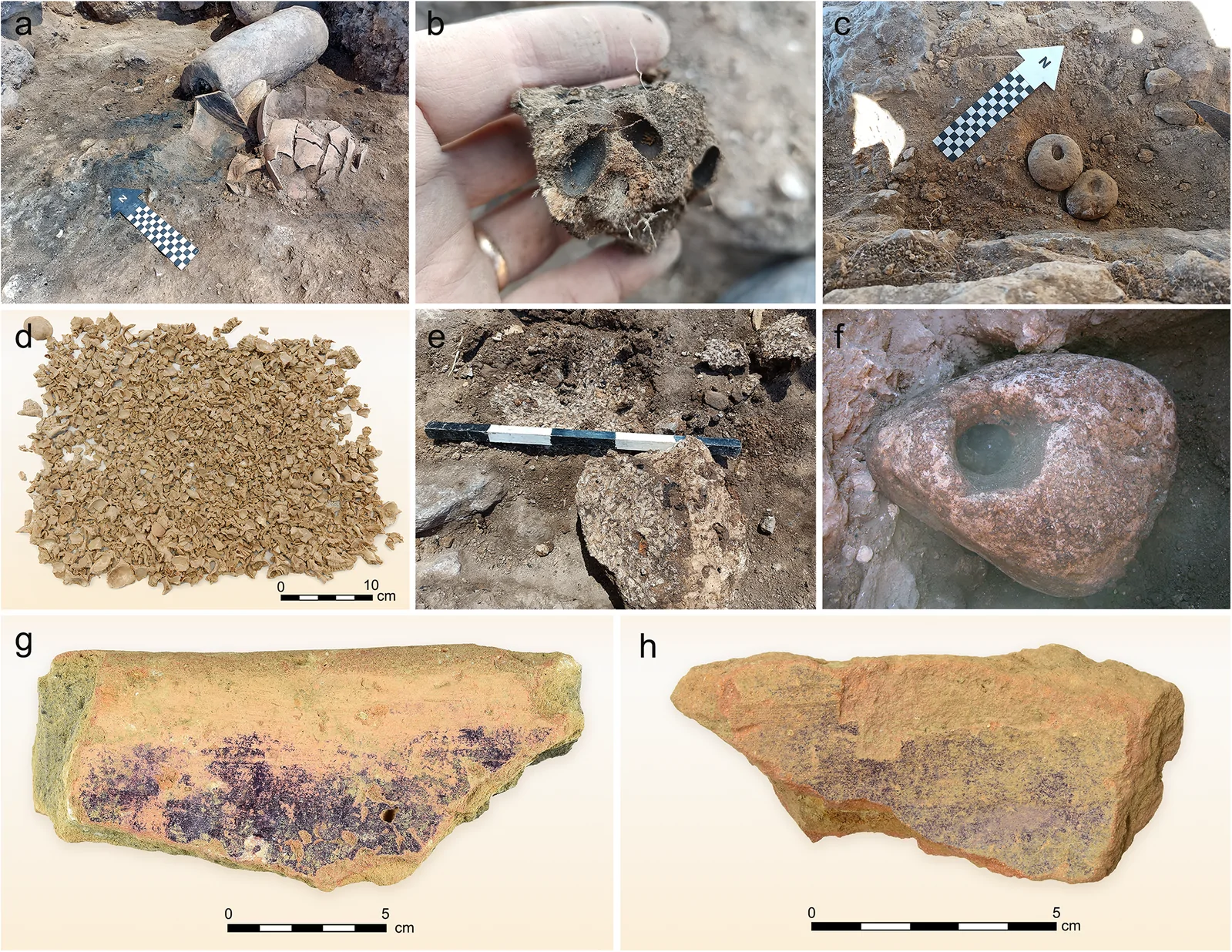
Selected finds from the Eastern (a–c) and Western (d–h) spaces. **a)** Phoenician transport jars (TJ2; Basket 23A-1082) *in situ* alongside an olive crushing stone (Basket 23A-1083); b) sediment with negative impressions of olives (Basket 23A-1096); c) loom weights (Basket 23-1084); d) pile of crushed Muricidae shells (Locus 23A-078); e) putative non-carbonized olive pits in sediment (Basket 23A-1126); f) olive press weight with hole and olive impressions; g-h) Purple-dye vat rim (Basket 13A-2020); and a body fragment (Basket 13A-2193).

**Fig 6:**
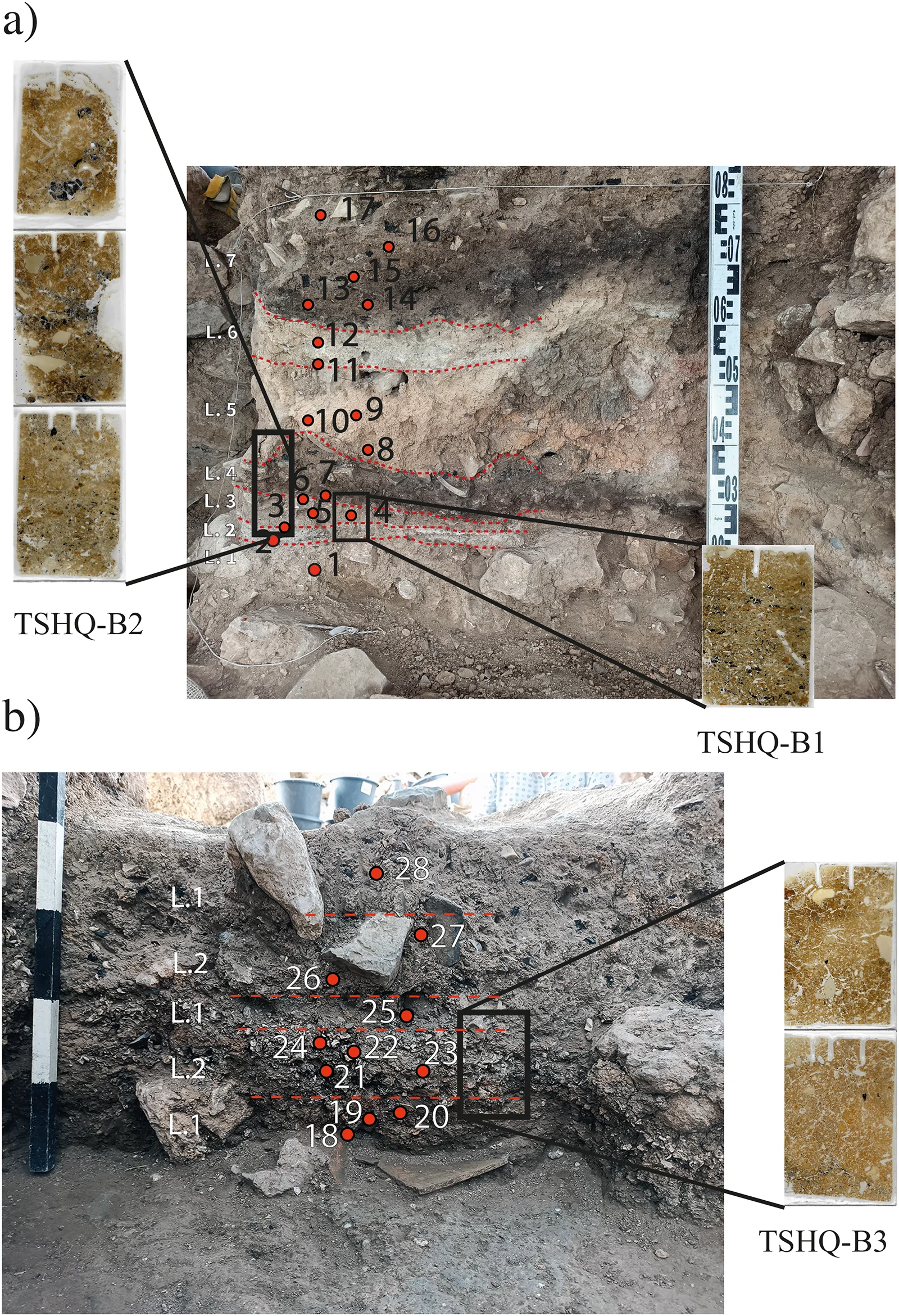
Location of micromorphological blocks from the Eastern Space cross-section (a) and the Western Space cross-section (b). The red dots represent the location of bulk samples.

As part of conservation and development efforts at Tel Shiqmona by the Israel Nature and Parks Authority, a section that threatened to collapse was excavated in 2023 (Fig 4). The Iron Age remains presented here are securely identified as part of the site-wide Stratum 11. This determination relies on the ceramic finds, the presence of a well-defined destruction layer characteristic of this stratum, and the elevations of walls and floors. Most crucially, it is based on the direct continuation of walls extending from Elgavish’s excavations through Bar’s excavations into the current excavation area (Fig 2). Stratum 11 is parallel to Bar’s Strata A6 and B1 [60, 61]. The excavated area is partitioned into two primary spaces by wall W23A-034, which features an opening that provides a functional link between the two (Fig 4):

*The Eastern Space* (Fig 4; brown): Located in the northeastern part of the excavated area and measuring 2 x 2 m, this area is delimited by later walls to the east and north (not illustrated; the plan depicts only pertinent Iron Age features), by the excavation limit to the south, and by Wall W23A-034 to the west. This area produced restorable Phoenician transport jars (TJ2; for the type see [62]), found *in situ* alongside a crushing/roller stone for olives and sediment embedded with imprints of olives (Fig 5a–b). Similar roller stones have been documented in olive-press contexts at other Iron Age sites in the Southern Levant, such as three examples from Tel Batash (Timnah [63: 246–247, Pl. 62:1–3]) and one from Tel Kinrot [64: Pl. 109:10]. Two loom weights were also recovered in a primary, *in situ* context (Fig 5c), pointing to possible textile-related activity in this space. Although few in number, their scarcity is most plausibly taphonomic rather than original: a massive later Byzantine wall cut directly through the adjacent area and probably truncated the associated assemblage, removing the bulk of any related finds. The northeastern section of this space, containing the sediment accumulations of Stratum 11, was preserved for micro-archaeological sampling (Fig 6a).

*The Western Space* (Fig 4; blue): Located to the west of and adjacent to the Eastern Space, and measuring approximately 3.5 x 2 m. This area is delimited by later wall to the north (not illustrated; as noted above, the plan depicts only pertinent Iron Age features), by the excavation limit to the west, by walls W23A-010 and W23A-028 to the south, and by wall W23A-034 to the east. This area produced distinct industrial waste. Key finds include a pile of crushed *Muricidae* shells (Fig 5d), and dozens of putative non-carbonized olive pits within the sediment (Fig 5e). These remains were found adjacent to an olive press weight bearing dozens of putative uncarbonized olive pits preserved on its surface (Fig 5f) [60:563]. Notably, a purple-dye vat rim and a vat body fragment, both displaying vivid dye stains (Fig 5 g, h), were also recovered in the near vicinity during Bar’s 2013 excavations from the Stratum 11 fills. A bulk was maintained in the center of the space to sample the sediments accumulated within (Fig 6b).

### Sample collection and laboratory analysis

A total of 28 bulk sediment samples and three block samples were collected, representing all macro-stratigraphic layers and their respective vertical and horizontal limits. All sediment samples and their respective contexts were documented according to the methodology outlined by [37].

In the Eastern Space, 17 bulk sediment samples were collected from the main section. One of them was taken from below the Stratum 11 main surface—beneath a row of stones that may have served as a foundation for compacted earthen floors. It serves as a reference for the area’s sedimentological composition prior to Stratum 11, most plausibly reflecting leveling or foundation preparation rather than a primary occupation deposit. Two undisturbed monolithic sediment block samples were collected just above Stratum 11 (Fig 6).

In the Western Space, 11 bulk samples were collected from the section, along with one undisturbed monolithic sediment block sample taken from the lower excavated layer for micromorphological analysis (Fig 6).

The bulk sediment samples were described and analyzed using various laboratory techniques, including Fourier Transform Infrared (FTIR) spectroscopy, to determine the mineral composition of the sediments [41], identify thermal signatures of altered clay [65], and detect the transformation of aragonite shells into calcite due to exposure to elevated temperatures (see review in [66]). A total of 30 shell fragments from the Western Space were analyzed using FTIR. Micro-remains quantification was performed on all bulk sediment samples using established protocols for phytoliths [67] and wood ash pseudomorphs and dung spherulites [68]. Phytolith identification was conducted on six selected samples—four from the Eastern Space and two from the Western Space. Morphotype analysis followed the International Code for Phytolith Nomenclature (ICPN; [69]). The classification of morphological groups, distinguishing different plant organs (i.e., phytoliths typical of grass leaves/stems versus those of inflorescences), was based on the following works [70–73: Appendix 3, 74,75]. Finally, six thin sections were prepared from the undisturbed monolithic sediment block samples (TSHQ-B1, 2, and 3) for micromorphological analysis and documented using standard micromorphological terminology [76, 77].

## Results

### The eastern space

From bottom to top, the following stratigraphic layers were observed in the field (Table 1). Apart from Layer 1 (under the main surface Stratum 11), all samples were collected from the fills accumulated during the Iron Age IIB. Only Layers 2–5 were sampled for micromorphological analysis (Table 2, Fig 6).

**Table 1:** Sediment collected from Eastern and Western spaces, showing results of bulk analyses. Mineralogical composition was determined by FTIR spectroscopy: Cl = Clay, (a) = altered by heat, (u/a) = unaltered by heat, Qz = quartz, Ca = calcite, CHAP = carbonated hydroxylapatite, Ar = aragonite. Micro-remains are expressed in million/1 gr of sediment.

Section	Layer	Sample No.	Description	FTIR	Dung spherulites	Ash pseudomorphs	Phytoliths
Eastern space	Layer 1	TSHQ-1	Grayish-brown sediment (control)	Cl(a/u), Ca, Qz	0.57	0	1.37
	Layer 2	TSHQ-2	Light brown sediment with shells and bones	Cl(a/u), Ca, Qz, CHAP	1.82	1.88	3.39
		TSHQ-3	Light grey sediment	Cl(a/u), Ca, Qz, CHAP	1.87	1.92	1.75
		TSHQ-4	Brown sediment	Cl(a/u), Qz, Ca	2.18	1.63	0.8
		TSHQ-5	Light grey sediment	Cl(a/u), Ca, Qz, CHAP	2.53	2.73	2.35
	Layer 3	TSHQ-6	Dark brown sediment with in ceramics and stones	Cl(a/u), Ca, Qz	4.48	1.33	1.46
	Layer 4	TSHQ-7	Charcoal layer	Charcoal, Cl(a/u), Ca, Qz	–	–	–
	Layer 5	TSHQ-8	Orange sediment	Cl(a/u), Ca, Qz	1.11	0	1.7
		TSHQ-9	Orange sediment	Cl(a/u), Ca, Qz	0.90	0	0.48
	Layer 6	TSHQ-10	Grey sediment	Cl(a/u), Ca, Qz	3.55	0	7.11
		TSHQ-11	Grey sediment	Ca	4.10	2.19	5.46
		TSHQ-12	Brown loose sediment	Ca, Ar, Cl(a), Qz, CHAP	2.38	1.84	5.92
	Layer 7	TSHQ-13	Black loose sediment	Cl(a), Ca, Qz, CHAP	12.91	0	5.01
		TSHQ-14	Dark brown sediment rich in chalk inclusion and roots	Cl(a/u), Ca, Qz, CHAP	4.75	0	3.76
		TSHQ-15	Dark brown sediment rich in chalk and shells inclusions and roots.	Cl(a/u), Ca, Qz, CHAP	2.14	0	3
		TSHQ-16	Dark brown sediment rich in chalk and shells inclusions as well as roots	Cl(a/u), Ca, Qz, CHAP	3.25	0	3.76
		TSHQ-17	Dark brown sediment rich in chalk, shells, and ceramics inclusions as well as roots	Cl(a/u), Ca, Qz, CHAP	0.71	0	1.14
Western space	Layer 1	TSHQ-18	Brown sediment	Cl(a/u), Ca, Qz, CHAP	0.46	0	2.58
		TSHQ-19	Brown sediment rich in shells (Murex)	Cl(a/u), Ca, Qz, CHAP	0.48	0.21	2.16
		TSHQ-20	Brown sediment rich in shells (Murex)	Ca, Ar, Cl(a/u), Qz	0.78	0.28	3.53
	Layer 2	TSHQ-21	Red sediment with white inclusions	Ca, Ar, Cl(a/u), Qz	0.64	0	1.45
		TSHQ-22	Light brown layer rich in shells (Murex)	Ca, Ar, Cl(a/u), Qz, CHAP	0.34	0	3.47
		TSHQ-23	Light brown layer rich in shells (Murex)	Ca, Ar, Cl(a/u), Qz, CHAP	0.28	0	3.31
			Black inclusion	Charcoal			
		TSHQ-24	Light brown sediment	Ca, Ar, Cl(a/u), Qz, CHAP	0.37	0	2.96
			Red inclusion	Cl(a), Ca, Qz			
	Layer 1	TSHQ-25	Brown sediment with black and White inclusion	Ca, Ar, Cl(a/u), Qz	1.24	2.63	1.72
			Black inclusion	Charcoal			
			White inclusion	Ca			
	Layer 2	TSHQ-26	Light brown (Clay)	Cl(a/u), Ca, Ar, Qz	0.37	0	1.95
		TSHQ-27	Light brown with black inclusion (inclusion)	Cl(a/u), Ca, Qz	0.46	0	3.53
			Black inclusion	Charcoal			
	Layer 1	TSHQ-28	Brown sediment	Cl(a/u), Ca, Ar, Qz, CHAP	0.81	1.49	2.37

**Table 2:** Micromorphological description (terminology following Stoops 2003) and interpretation of the studied micromorphological samples. The layers identified along the sedimentary sequences in the cores are numbered from bottom to top (for context, see Fig 6).

Context	Macroscopic	Micromorphological observations	Interpretation
Eastern space	Layer: 2	**Microstructure:** complex, massive and vughy **Voids:** vughs, channels voids (~20%) **C/f related distribution:** Porphyric **Groundmass:** two main types of groundmass are observed (~20%). 1) yellow groundmass dominated by dung spherulites. 2) grey groundmass dominated by ash pseudomorphs **Coarse inclusions:** Coarse and medium grain size of Charcoal (~30%) shells (5%) and calcareous stones (limestone, beachrock or Kurkar) (10%). Coarse medium size grains of bones, clay lumps, heated clay chunks (pottery?) (~10%). Sub-rounded medium to fine grain size of quartz, Feldspar, iron-manganese nodules (<5%).	Activities associated with Stratum 11 are characterized by evidence of animal husbandry, reflected in abundant dung spherulites and micro-trampling features, together with recurrent maintenance activities resulting in high concentrations of wood ash pseudomorphs and sporadic combustion events.
	Layer 3	**Microstructure:** Complex: Massive, vughy (highly porous and poorly sorted) **Voids:** vughs, channels, packing voids (~30%) **C/f related distribution:** Porphyric **Groundmass:** brown groundmass (~10%). **Coarse inclusions:** Coarse and medium grain size of Charcoal (~15%) shells (~5%) and calcareous stones (limestone, beachrock or Kurkar) (~15%). Coarse medium size grains of bones, clay lumps, heated clay chunks (pottery?) (~20%). Sub-rounded medium to fine grain size of quartz, Feldspar, iron-manganese nodules (<5%).	Final phase of activity within Stratum 11 associated with animal husbandry and maintenance practices, resulting in a more heterogeneous deposit characterized by large inclusions and mixed sediments rather than finely laminated microstratigraphy.
	Layer 4	**Microstructure:** Vughy (highly porous and poorly sorted) **Voids:** vughs, channels, chamber voids (~40%) **C/f related distribution:** Porphyric **Groundmass:** complex groundmass with dark brown, light yellow and orange color together with black coloration due to the amount of charcoal (~5%). **Coarse inclusions:** Coarse and medium grain size of Charcoal (~40%) and calcareous stones (limestone, beachrock or Kurkar) (~5%). Coarse medium size grains of bones, clay lumps, heated clay chunks (<5%). Sub-rounded medium to fine grain size of quartz, Feldspar, iron-manganese nodules (<5%).	Stratum 11 destruction layer. Interpreted to form due to *in situ* conflagration, resulting in collapsed roof remains (wood beams, thatch and mud), mixed with activity residues and degraded earth-based construction materials.
	Layer 5	**Microstructure:** complex: massive and vughy **Voids:** packing voids, vughs (~20%) **C/f related distribution:** Porphyric **Groundmass:** the ground mass is dominated by a light brown color the b/fabric is speckled (~30%). **Coarse inclusions:** Coarse and medium grain size of Charcoal (~15%) shells (~10%) and calcareous stones (limestone, beachrock or Kurkar) (~10%). Coarse medium size grains of bones, clay lumps, heated clay chunks (pottery?) (~10%). Sub-rounded medium to fine grain size of quartz, Feldspar, iron-manganese nodules (<5%).	Post-destruction layer formed due to continuous degradation of mudbrick walls (a distinct depositional unit not associated with any single stratum); followed by infilling of local site sediment composed of archaeological materials and marine organisms, but without substantial quantities of dung spherulites or wood ash pseudomorphs.
Western space	Layer 1	***Microstructure***: complex: Massive and vughy microstructure ***Voids:*** Vughy and planar voids (~20%). ***C/f related distribution:*** Porphyric ***Groundmass:*** PPl dark brown (~20%) B-fabric: Speckled occasionally grano striated ***Coarse inclusions:*** Quartz rounded medium sand and silt (~10%). Calcareous rounded coarse and medium sand grains as well as large number of angular shells fragments (~30%) Feldspar angular medium and fine sand grains >5% Basalt coarse and medium sand subrounded <5% Coarse and medium sand anthropogenic intrusion: pottery fragments, clay lumps and charcoal (~10%). Occasionally flint and bone fragments are also noted	Infill of local sediment, enriched in shells and burnt material
	Layer 2	***Microstructure***: Complex: Massive and vughy microstructure ***Voids: V***ugh and planar voids **(**~20%). ***C/f related distribution:*** Porphyric ***Groundmass:*** PPl light brown (~25%) B-fabric: Speckled occasionally grano- striated ***Coarse inclusions:*** Quartz rounded medium sand and silt 10- ~ 15%. Calcareous rounded coarse and medium sand grains as well as large number of angular shells fragments (~30%) Feldspar angular medium and fine sand grains and basalt coarse and medium sand subrounded <5% Coarse and medium sand anthropogenic intrusion: pottery fragments, clay lumps and charcoal (~5%). Occasionally flint and bone fragments are also noted	Infill of local sediment enriched with shells

**Fig 7:**
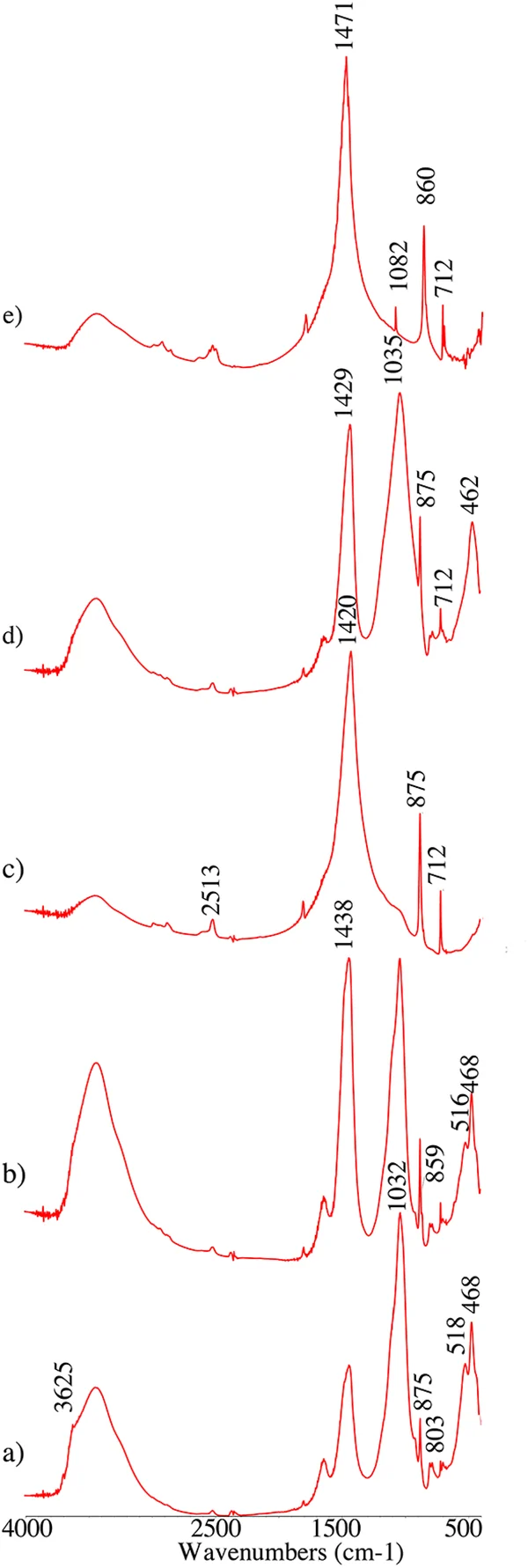
Representative FTIR spectra. **a)** General matrix of the Eastern Space composed of unheated clay (3695, 3625, 1032, 915, 525, 460 cm^−1^), calcite (2500, 1420, 875, 712 cm^−1^), and minor traces of quartz (noted mainly in the double peak around 798, 779, cm^−1^); b) General matrix of the Western Space composed of unheated clay, calcite, and aragonite (1476, 1083, 857, 713, 700 cm^−1^); c) White inclusion from the Western Space composed of calcite; d) Red inclusion from the Western Space composed of heated clay (note the absence of peaks at 3695, 3625, 915 cm^−1^ and the shift of the main silicate peak towards 1035 cm^−1^), calcite, and minor traces of quartz. e) Shell composed of aragonite from the Western Space.

**Fig 8:**
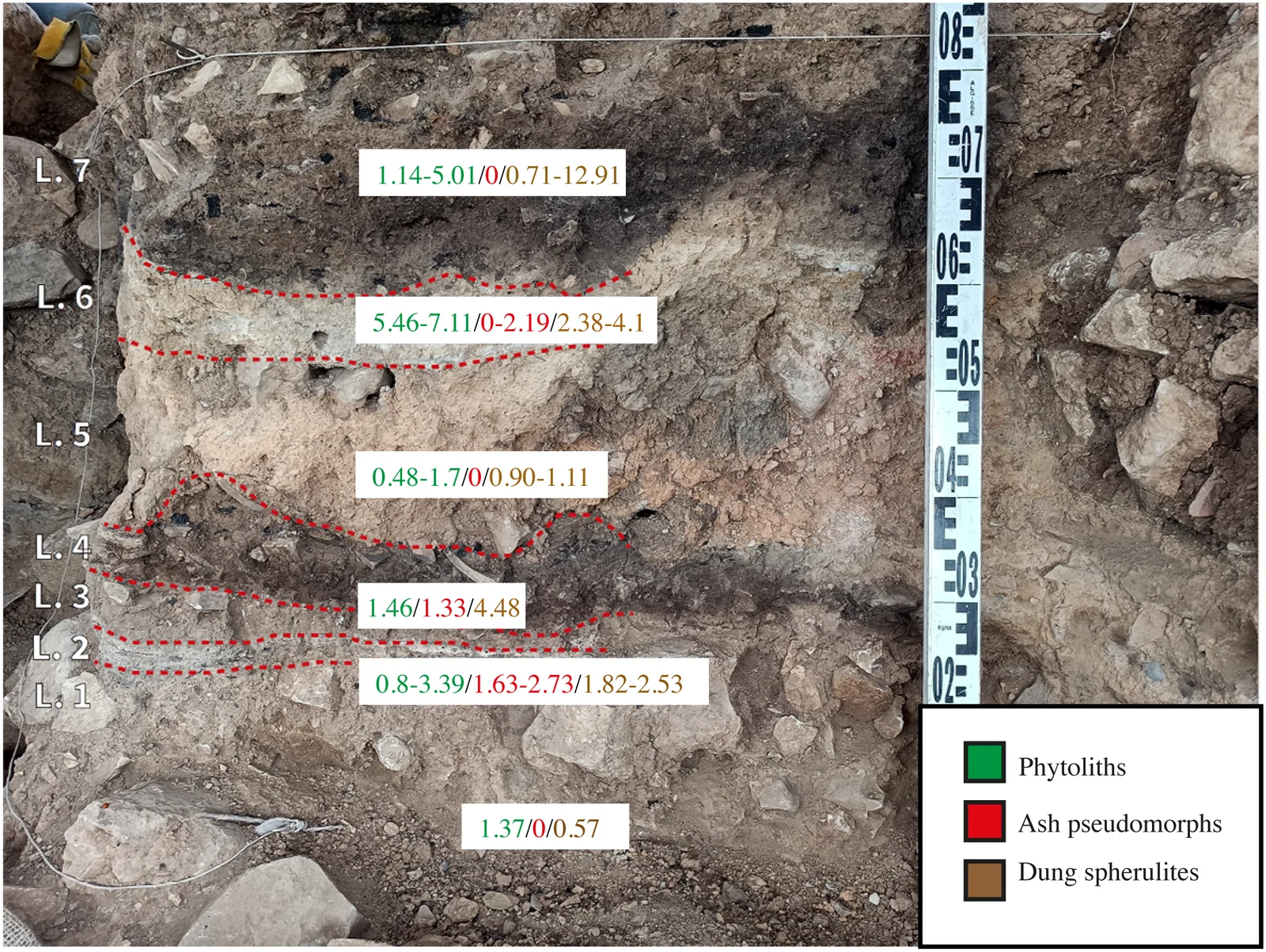
Distribution of micro-remains in the Eastern Space. The number of micro-remains is expressed in million per gram of sediment, phytoliths (green), ash pseudomorphs (red), and dung spherulites (brown).

**Fig 9:**
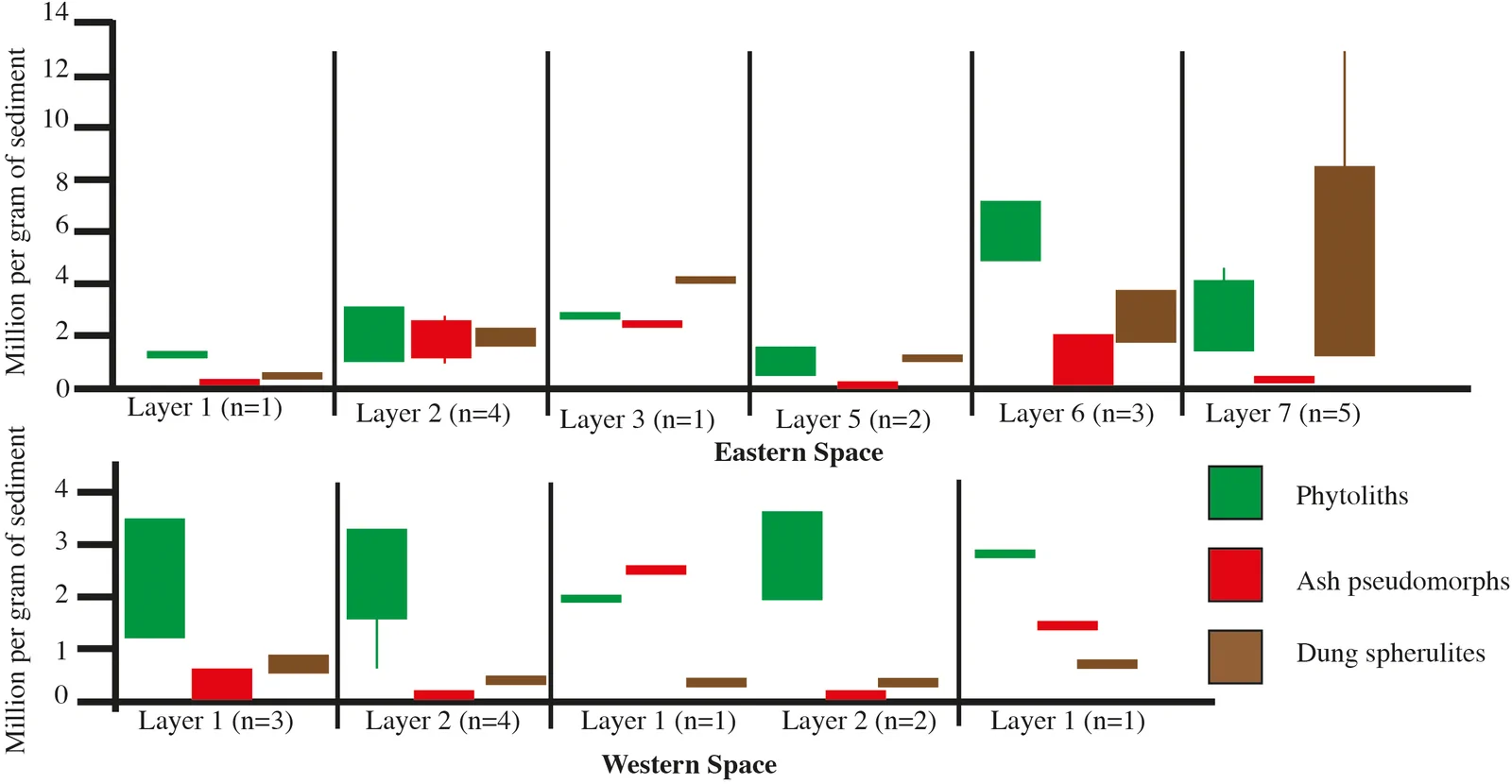
Distribution of micro-remains in the Eastern Space and the Western Space. Phytoliths (green), ash pseudomorphs (red), and dung spherulites (brown).

Layer 1: A gray-brown sediment containing a variety of unsorted stones, exposed beneath the Stratum 11 compacted earthen floors. This accumulation predates the Stratum 11 main occupation. Mineralogically, the sediment (TSHQ-1) is composed of unaltered clay, calcite, and quartz (Fig 7a). Micro-remains were found in relatively low amounts, with 0.57 million dung spherulites and 1.37 million phytoliths per gram of sediment (Figs 8, 9).

**Fig 10:**
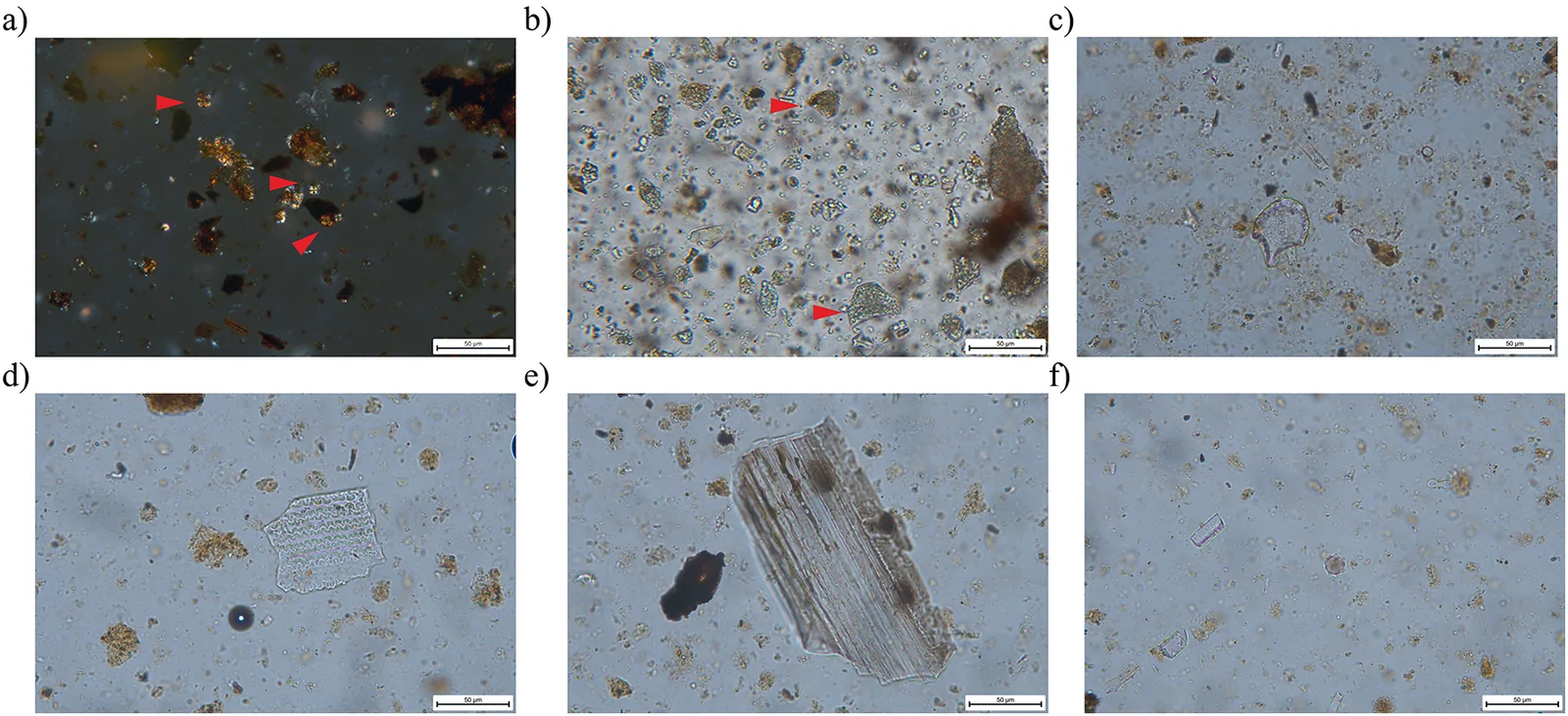
Micro-remains. **a)** Dung spherulites (red arrow), b) Ash pseudomorphs (red arrow). Phytolith morphologies: c) Bulliform cell cuneiform and short cell rondel, d) Long cell dendritic and long cell wavy, e) Parallelepiped elongate psilate, f) Spheroid echinate.

**Fig 11:**
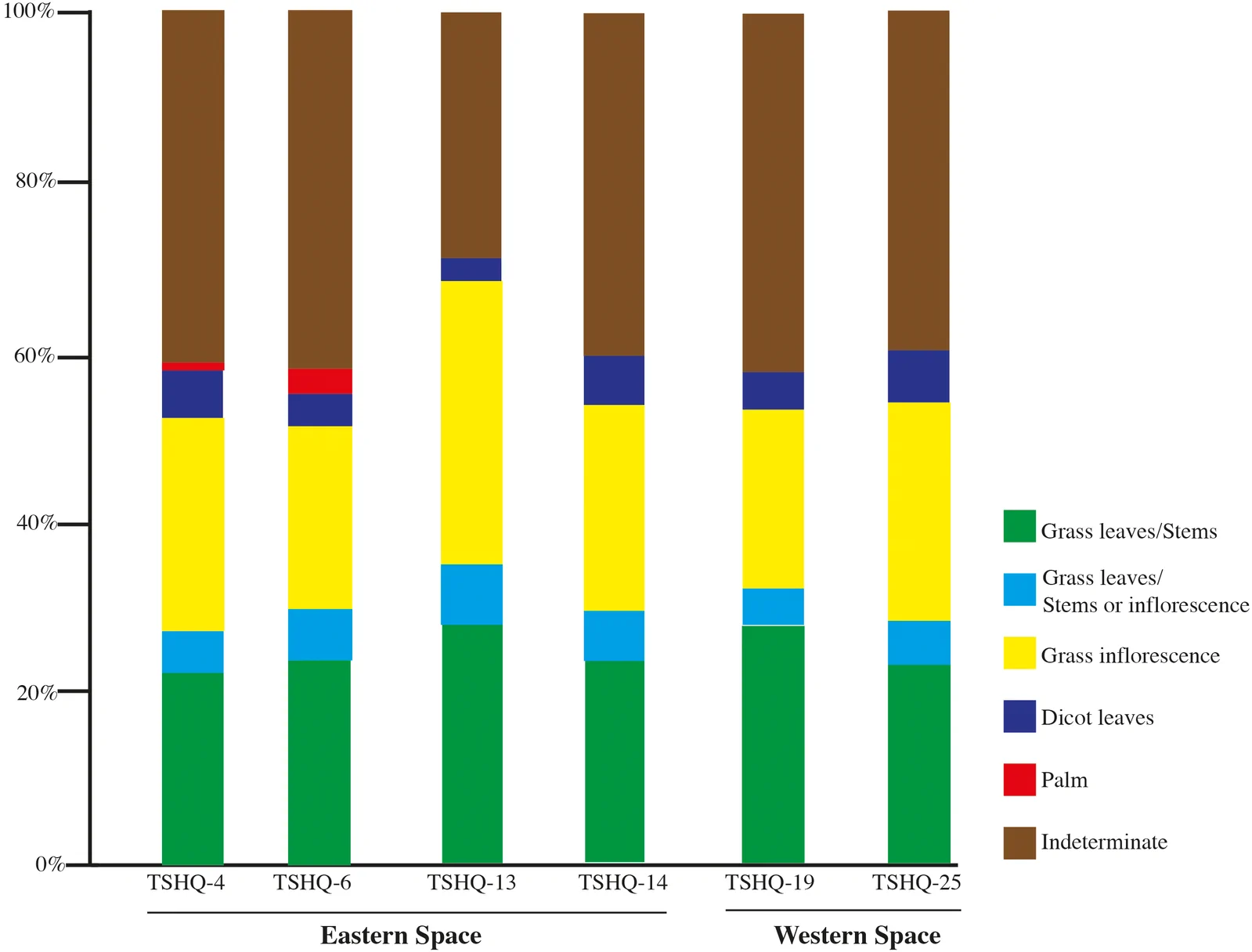
Phytolith morphologies found in sediment in the Eastern Space and the Western Space. All sediments include phytoliths from both monocotyledonous and dicotyledonous plants. The most prevalent phytoliths are from grasses, where all plant parts (leaves/stems and inflorescences) are represented in varying proportions.

**Fig 12:**
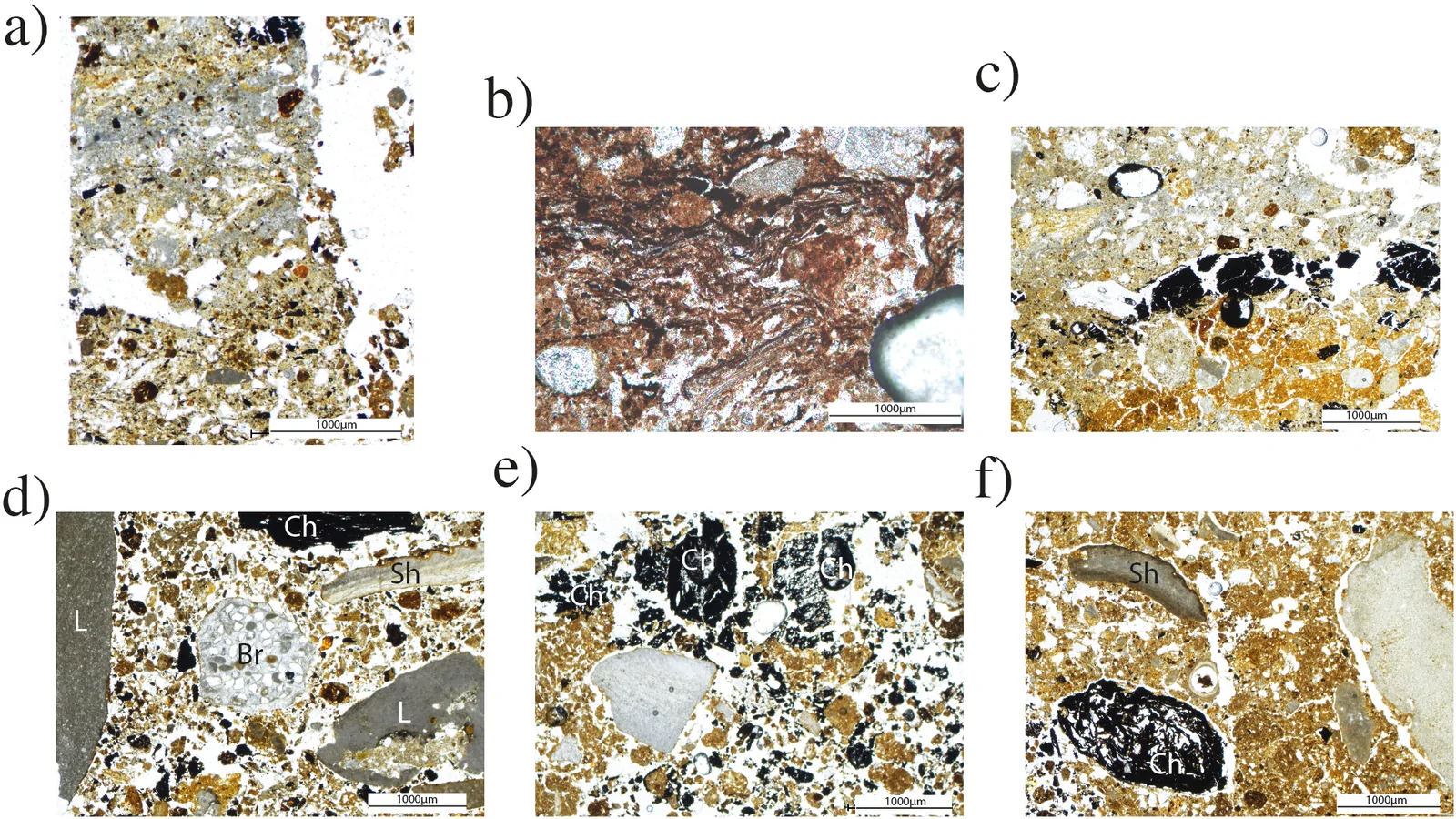
Microphotographs (PPL) of the Eastern Space. Layer 2: a) showing alternating laminae of light brown (dung-rich) and gray (cemented wood ash) sediment that characterized Layer 2. b) Trampling of micro-charcoal and phytoliths frequently found in Layer 2. Note charcoal and organic remains breaking *in situ*. c) A combustion feature found within Layer 2. Note the rubified earth substrate (red) overlain by a layer of charcoal (black) with a layer of wood ash at the top (gray). d) Groundmass of Layer 3 rich in coarse sand inclusions of beachrock (br), limestone (L), charcoal (Ch), and shells (Sh), and presenting a vughy microstructure, highly porous and poorly sorted. e) Groundmass of Layer 4 sharing similar microstructural characteristics as Layer 3 (panel d), but with dominance of charcoal (Ch). f) Groundmass of Layer 5. Note the dominance of a massive microstructure and the abundance of coarse sand inclusions (i.e., charred organic matter (Ch), shells, and calcareous stones).

Layer 2: Layer 2 consists of two brown laminae (TSHQ-2, 4) and two gray laminae (TSHQ-3, 5) located above the unsorted stones of Layer 1. These laminae are mainly composed of unaltered clay, calcite, and quartz, similar to the control and other layers (Fig 7a). The layer is rich in micro-remains, with 1.82–2.53 million dung spherulites, 0.80–3.39 million phytoliths, and 1.63–2.73 million ash pseudomorphs per gram of sediment (Figs 8–11). Micromorphologically, Layer 2 was sampled in both blocks (TSHQ-B1 and 2) (Table 1, Fig 6). The layer is composed of patchy thin laminae (1–3 mm thick) with brown and gray groundmass colors (~20%). The brown groundmass is rich in dung spherulites, overlain by a gray groundmass dominated by ash pseudomorphs (Fig 12a). The microstructure is complex, alternating massive microstructure and vughy microstructure. Vugh, chamber, and channels voids are frequent (~20%). Evidence of trampling, represented by *in situ* breakage of organic materials, occurs sporadically throughout the layer (Fig 12b). Additionally, *in situ* fire activity is identified in the uppermost part of TSHQ-B1, represented by indicative layering with a rubified layer, overlain by a charcoal layer, and an ash layer at the top (Fig 12c). The sequence continues with alternating brown and gray groundmass. The sediment is rich in coarse and medium sand-sized fragments of micro-charcoal and other organic remains (~30%). Clay lumps (~10%) are abundant, while coarse and medium sand-sized fragments of bone and angular flints are present in lower quantities (~5%). Other coarse and medium sand-sized fragments of micritic calcite and limestone, together with shells, are also noted (~10%), with low quantities of rounded quartz grains (~5%).

Layer 3: A brown layer rich in pottery sherds was identified. The sediment (TSHQ-6) is mineralogically composed of unaltered clay, calcite, and quartz. The layer is rich in micro-remains, with 1.46 million phytoliths, 4.48 million dung spherulites, and 1.33 million ash pseudomorphs per gram of sediment (Figs 8, 9). Micromorphologically, the layer is characterized by a dark brown groundmass (~10%) with a complex microstructure combining massive and vughy microstructure (highly porous and poorly sorted). Voids, chambers, and channels are frequent (~30%). Coarse sand-sized calcite inclusions (~15%) and shell fragments (~5%) are abundant. Coarse medium grains of archaeological materials (~20%) as well as charcoal and other organic materials (~15%) are embedded within this layer (Fig 12d). Sub-rounded medium to fine-grained quartz and feldspar were noted (~5%).

Layer 4 is characterized in the field by its black color, rich in charred material, and a high concentration of pottery. Mineralogically, the sediment (TSHQ-7) is composed mainly of unaltered clay, calcite, and quartz. The layer is rich in charcoal, and micro-remains were not extracted. Micromorphologically, Layer 4 has sporadic fragments of groundmass (~5%), a vughy microstructure, highly porous and poorly sorted. Vughs and chamber voids are frequent (~40%). The layer contains abundant large and micro-charcoal fragments (40%). Coarse and medium grain size of calcareous stones (i.e., kurkar) are present (~5%). Anthropogenic materials include bones, clay lumps, and flint (~5%). Sub-rounded medium to fine-grained quartz (~5%) are observed. The transition between Layer 3 and Layer 4 presents a diffuse contact in which charred materials from Layer 4 are mixed with Layer 3 (Fig 12e).

Layer 5: Above Layer 4, an orange sediment is noted, scarce in archaeological material. Mineralogically, the sediment (TSHQ-8, 9) is composed of unaltered clay, calcite, and quartz (Fig 7a). It lacks ash pseudomorphs but contains 0.48–1.70 million phytoliths and 0.90–1.11 million dung spherulites per gram of sediment, indicating lower overall micro-remains, similar to those in Layer 1 which served as a control sample (Table 1, Figs 8–9). Micromorphologically, Layer 5 has a dark brown groundmass (~30%) with a complex microstructure between massive and vughy microstructures. The sediment becomes compacted in the upper part of the thin-section (Fig 12f). Vughy voids are less dominant (~20%). The layer contains coarse and medium sand inclusions, as well as calcareous stone fragments (~10%), charcoal fragments (~15%), and angular medium sand shell fragments (10%). Other anthropogenic materials include clay lumps, angular flint, and bone (~10%). Sub-rounded, medium- to fine-grained quartz sand grains are noted (~5%).

Layer 6: Layer 6 contains orange, black, and gray sediments. The three samples from this layer (TSHQ-10, 11, and 12) have different mineralogical compositions: TSHQ-10 is composed of unaltered clay, calcite, and quartz; TSHQ-11 is composed mainly of calcite; and TSHQ-12 is composed of calcite, aragonite, clay. and quartz. All samples have high concentrations of micro-remains: 5.46–7.11 million phytoliths, 2.38–4.10 million dung spherulites, and 0–2.19 million ash pseudomorphs per gram of sediment (Table 1, Figs 8–9).

Layer 7: At the top of the section, a black layer of sediment is observed. Mineralogically, this layer (TSHQ-13, 14, 15, 16, 17) is composed of clay, calcite, quartz, and hydroxyapatite. The sediment is rich in phytoliths (1.14–5.01 million per gram of sediment) and dung spherulites (0.71–12.91 million per gram of sediment), but lacks ash pseudomorphs (Table 1, Figs 8–9).

Samples (TSHQ-4, 6, 13, 14) from the layers with higher phytolith concentrations (Layers 2, 3, and 7) were selected for phytolith identification. All samples are dominated by grass leaves, stems, and inflorescence (51–68%) and wood/bark phytoliths (3–5%). Most samples include dendritic long cells (7–10%), which are associated with the inflorescence of domesticated cereals (i.e., wheat or barley) [78: Appendix I, 79]. While this is the general composition of all samples, TSHQ-6, representing Layer 3 (located below Layer 4, a large charcoal-rich layer), also contains a small amount of spheroid echinate morphotypes (3%), which are associated with palm [80] (Table 3, Figs 10–11).

**Table 3:** Phytolith morphotypes identified in selected samples. The percentage of dendritic long cells appears in bold italics.

Morphotype	TSHQ- 4	TSHQ- 6	TSHQ- 13	TSHQ- 14	TSHQ- 19	TSHQ- 25
Platelet (epidermal skeleton)	1	1	0	0	0	0
Irregular rugulate	7	8	6	9	9	11
Spheroid psilate	0	0	0	0	1	1
Long cell dendritic	15	19	22	15	14	16
Long cell echinate	22	16	19	18	25	20
Long cell verrucate	7	2	5	3	0	0
Long cell wavy	8	12	21	8	13	17
Papillae	0	1	7	0	2	1
Bulliform cell cuneiform	4	8	1	3	2	3
Bulliform cell parallelipedal	1	1	11	3	0	1
Cylindroid psilate	41	46	37	42	51	42
Epidermal appendage [prickle]	1	2	7	3	1	3
Long cell sinuous	0	0	7	8	0	0
Short cell bilobate	5	6	5	3	4	7
Short cell polylobate	0	0	1	0	2	0
Short cell saddle	1	0	5	0	1	0
Short cell rondel	3	6	5	6	5	2
Short cell rondel tower	1	2	0	0	1	2
Parallelepiped blocky psilate	43	41	30	44	47	37
Stomata	0	2	0	0	1	0
Parallelepiped elongate psilate	43	56	34	44	42	47
Parallelepiped elongate rugulate	0	0	0	0	0	0
Spheroid echinate	1	7	0	0	0	0
**Total**	**204**	**236**	**223**	**209**	**221**	**210**
**Grass Leaves/Stems**	23%	24%	28%	28%	24%	23%
**Grass Leaves/Stems or Inflor**	5%	6%	7%	4%	6%	5%
**Grass Inflor**	25%	21%	33%	21%	24%	26%
**Dicot Wood/Bark**	5%	4%	3%	4%	6%	6%
**Palm**	0%	3%	0%	0%	0%	0%
**Indeterminate**	41%	42%	29%	42%	40%	40%
***(Dendritic %)***	***7%***	***8%***	***10%***	***7%***	***6%***	***8%***

### The Western Space

Macroscopically, a succession of brown (n = 3) and light brown (n = 2) alternating sediment layers is observed in the accumulation fills of Stratum 11 in the Western Space. Two main stratigraphic layers were distinguished (Table 1 and 2, Fig 6).

**Fig 13:**
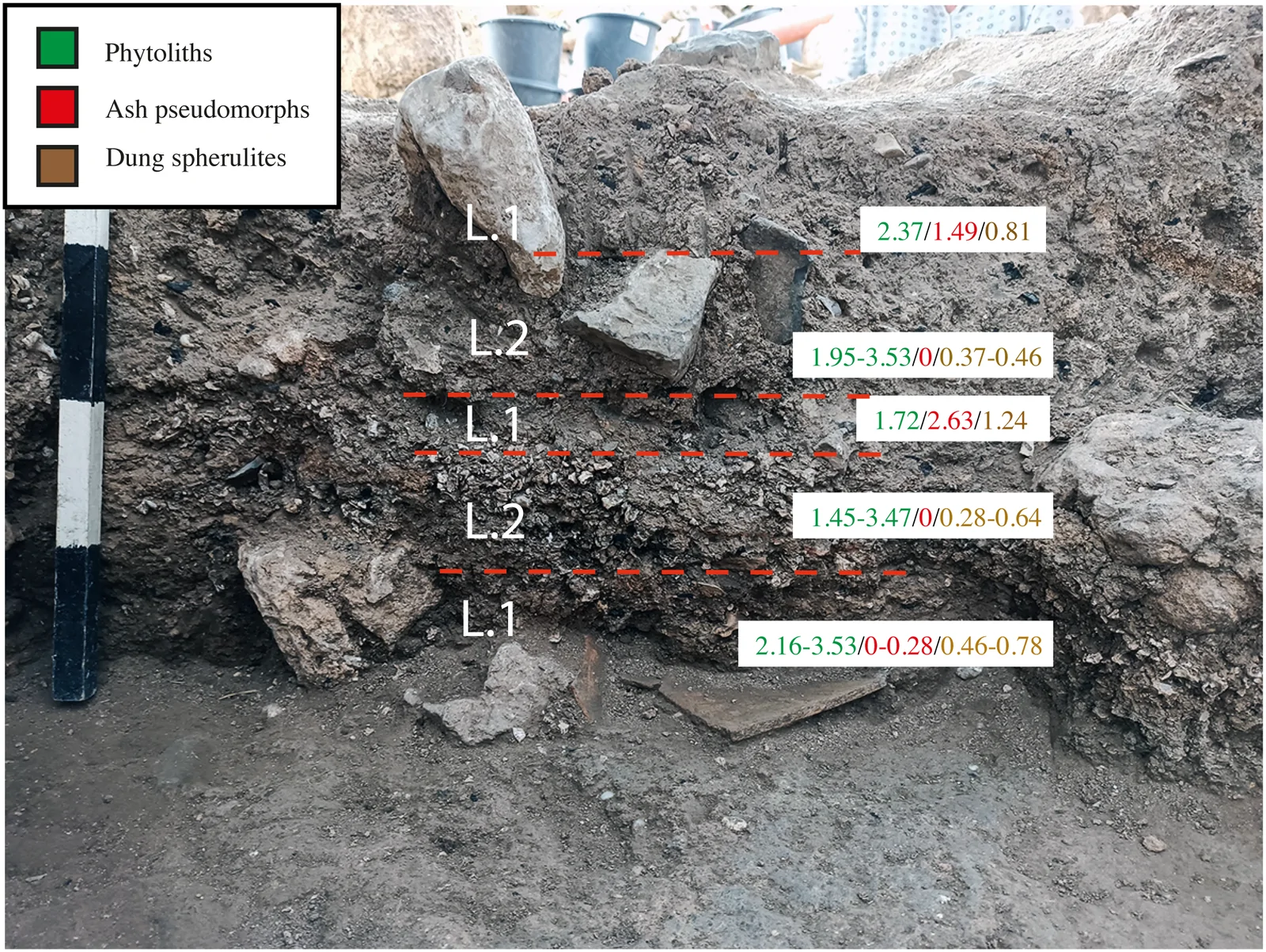
Distribution of micro-remains in the Western Space. The number of micro-remains is expressed in millions per gram of sediment, phytoliths (green), ash pseudomorphs (red), and dung spherulites (brown).

Layer 1: This layer is composed of brown sediment (TSHQ-18,19,20,25,28). The lower part of Layer 1 is richer in shell fragments. The mineralogical composition consists of unaltered clay, calcite, aragonite, quartz, and occasional carbonated hydroxyapatite (Fig 7b). The brown layers are generally richer in black and white inclusions. The black inclusions are mostly associated with charcoal, and white inclusions are composed of geogenic calcite (Fig 7c). Shells are composed of aragonite. The layer contains phytoliths (1.72–3.53 million per gram of sediment) and ash pseudomorphs (0–2.63 million per gram of sediment). Dung spherulites range between 0.46–1.24 million per gram of sediment (Table 1, Figs 9–10, Fig 13).

**Fig 14:**
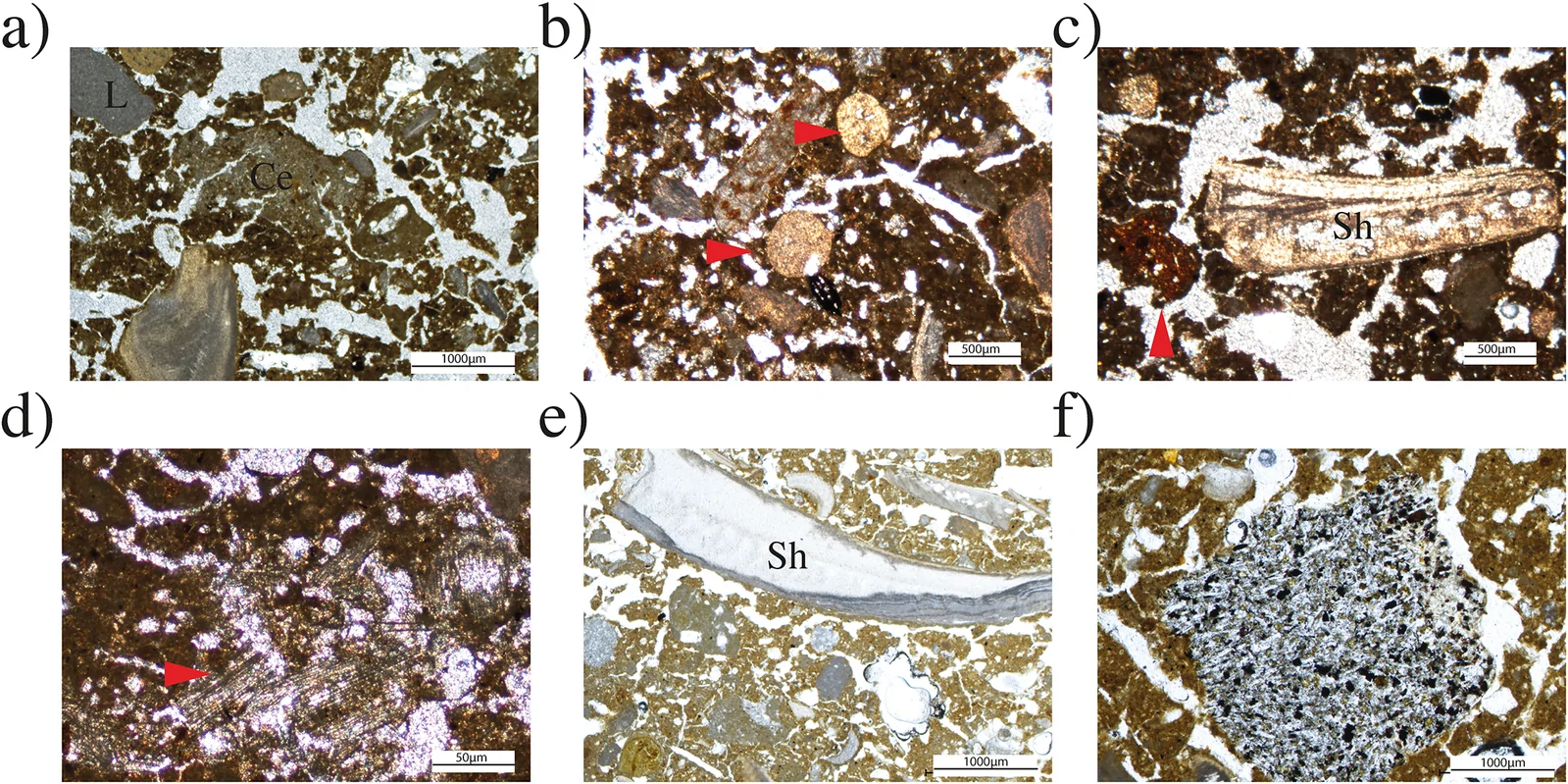
Microphotographs of the Western Space. Layer 1. a) dark brown groundmass rich in cemented calcareous areas (Ce) and limestone fragments (L). b) Possible phosphate nodules (red arrow) within the dark brown groundmass. c) Large fragment of shells (Sh), and rubified clay lump (red arrow) within Layer 1. d) Phytoliths (red arrow) within groundmass in Layer 1. e) Light brown groundmass with large fragments of shells (Sh) and rounded medium-grain calcareous stones associated with Layer 2. f) Basalt inclusion.

Micromorphologically, the layer is characterized by a dark brown groundmass (~20%). The microstructure is complex, combining massive and vughy microstructures. Vugh and planar voids composed up to (~20%) (Fig 14a). The groundmass is rich in ash pseudomorphs and micro-charcoal, possible burnt clay lumps, and phosphate nodules (~10%) (Fig 14 a, b, c, d). Shells are frequently observed, usually medium sand size, and angular in shape (~30%). Subrounded coarse and medium sand inclusions of basalt fragments are frequent (~5%) (Fig 14e). Rounded medium sand and silt grains of quartz are found (~10%). Medium sand-sized fragments of shell and bone are also present (~5%).

Layer 2: Macroscopically, Layer 2 is composed of a light brown sediment (TSHQ-21, 22, 23, 24, 26, 27). As in Layer 1, the lower part of Layer 2 is richer in shell fragments than the upper part. There are no major mineralogical differences between these sediments, which are composed of unaltered clay, calcite, aragonite, quartz, and occasional carbonated hydroxyapatite (Fig 7b). The shells are composed of aragonite. White, black, and red inclusions are frequent. The red inclusions are composed of altered clay (Fig 7d) (based on [65]), while the white inclusions are mainly composed of geogenic calcite (Fig 7c), and the black inclusions are related to charcoal fragments. The sediment contains a high number of phytoliths (1.45–3.53 million per gram of sediment). Dung spherulites (0.28–0.64 million per gram of sediment) are scarce, while ash pseudomorphs are absent in this layer (Table 1, Figs 9–10, 13).

Micromorphologically, the layer is characterized by a light brown groundmass with massive and vughy microstructure (~25%) (Fig 14f). Vughs and planar voids are dominant (~20%). Shell fragments and rounded coarse and medium sand grains of calcareous materials are also frequent (~30%) (Fig 14f). Rounded medium sand and silt grains of quartz are noted (~15%). Subrounded coarse and medium sand grains of basalt are found (~5%). Cemented ash pseudomorphs are not present, and other anthropogenic remains such as small to medium sand-sized fragments of flint and bone, phosphate nodules, clay lumps, and micro-charcoal are found in low amounts (~5%)

Two samples (TSHQ-19, 25) were selected for phytolith identification, one from each layer. The samples are dominated by grasses (54%) and wood/bark phytoliths (6%). All samples include dendritic long cells (6–8%) (Figs 9–11; Table 3)

## Discussion

### The stratigraphic sequence and activity patterns

The integration of macro-archaeological data with high-resolution micro-geoarchaeological analysis has enabled a detailed reconstruction of the life cycle and spatial use of a defined area in the industrial complex at Tel Shiqmona.

In the *Eastern Space,* the sequence begins with Layer 1, located beneath the main earthen floors of Stratum 11 (Figs 4 and 8). This layer, characterized by low concentrations of micro-remains (<2 million micro-remains per gram of sediment) and anthropogenic evidence, likely represents preparatory activity (i.e., leveling), possibly associated with laying the foundations for the first floor of Stratum 11 in the earthen surface. The floor and the activity accumulation upon it are not distinguishable as separate units and are represented together by Layer 2.

Layer 2 represents the initial deposits accumulated on the earthen floors. The microscopic record reflects evidence of animal husbandry. The sediments contained a high number of dung spherulites (2.53 million per gram of sediment), alongside clear indications of trampling of phytoliths and micro-charcoals (Fig 12b), providing clear evidence for livestock keeping. The maintenance of the area is visible in the micro-stratigraphy, which displays a cyclical alternation between dung-rich deposits and wood-ash laminations (Fig 12a), alongside sporadic burning events (Fig 12c). These can be interpreted as evidence of site maintenance, involving the spreading of ashes across animal penning surfaces to sanitize the area (e.g., [81]).

The sequence of activity continues through Layers 3 and 4, representing the ongoing life of the structure leading up to its final destruction. Like Layer 2, Layer 3 is rich in micro-remains, containing 4.48 million dung spherulites per gram of sediment, indicating the continuous presence of livestock. Layer 4 marks the terminal phase of Stratum 11: resulting from the destruction itself, in which burnt roof beams and thatch are mixed with the activity residues, left by the last phase of activity within the building, and earth-based construction materials (e.g., mudbrick walls). As an *in situ* destruction deposit it is an integral part of Stratum 11. In contrast to the underlying occupation deposits, it yielded no extractable micro-remains; micromorphologically it is dominated by abundant charcoal and coarse anthropogenic inclusions (bone, flint, and clay lumps) set within a porous, poorly sorted groundmass, a composition consistent with a destruction horizon rather than an occupation surface (see Destruction and Post-Abandonment Activity, below). It is within this same context, before destruction, that the macro-archaeological evidence for industry is most pronounced.

Key finds include an olive press roller stone and a concentration of olive pits preserved as compressed negatives within the sediment. Loom weights recovered from the destruction debris further suggest that textile-related activities also took place within this space. Consequently, the integration of micro- and macroscopic finds in the sequence of Layers 2–4 demonstrates that throughout the life of Stratum 11, the space served several functions: housing livestock, olive oil extraction, and textile production.

In the adjacent *Western Space*, the archaeological findings revealed a high abundance of crushed *Muricidae* shells alongside dozens of putative non-carbonized olive pits impressed into the sediment. These were found in close proximity to an olive press weight and purple-stained pottery (recovered in earlier excavations by Bar; see above), suggesting the area served as a multi-functional workshopfor purple-dye production and dyeing andfor olive processing. Microscopically, this industrial sequence is manifested in two alternating layers (Layer 1 and 2) rich in shell fragments. The distinction between the two layers lies in the abundance of firing residues: Layer 1 is rich in wood ash pseudomorphs, micro-charcoal, and heated clay lumps, while Layer 2 lacks wood ash pseudomorphs and contains significantly less charcoal. This suggests episodic dumping of discarded shells and fire residues, indicating that the use of fire was an integral part of the purple-dye production process (though not for directly heating the dye vats themselves; see below), as evidenced by the presence of marine mollusks in direct association with wood ash, charcoal, and heated clay.

#### Destruction and Post-Abandonment Activity.

The physical destruction of Stratum 11 is clearly marked by the nature of Layer 4 in the *Eastern Space*, which belongs to and marks the end of Stratum 11. This layer represents the sudden event of collapse, characterized by a charcoal-rich composition, an abundance of pottery vessels, a distinctive vughy (highly porous and poorly sorted) microstructure, and rich coarse anthropogenic inclusions (i.e., charcoal and bone fragments, angular flint, clay lumps). These deposits likely represent the collapse of the roof and its beams mixed with destruction debris, a distinct observation that aligns with findings from other areas of Stratum 11 excavated by Elgavish [57]. The presence of spheroid echinate phytolith morphotypes in Layer 3, associated with palm trees [80], further supports the interpretation of roof collapse composed of wood beams and thatch [82].

Layer 5 represents the physical collapse and degradation of the mudbrick walls [82]. It reflects lack of activity, and forms a distinct depositional unit in its own right. Layers 6 and 7 (Stratum 10) then indicate a shift in the use of space, a return to animal husbandry, as evidenced by the high concentration of animal dung spherulites (0.71–12.91 million per gram of sediment). The ceramics in these layers display characteristics of the Iron Age IIB horizon, albeit a later phase within this period, corresponding to the subsequent Stratum 10. These findings support the previous hypothesis that during Stratum 10, the site featured a single four-room house serving as a farmstead, while livestock roamed the remaining open areas of the mound [56].

### Synthesis: Seasonality and the industrial cycle

As outlined in the Introduction, multi-functionality and seasonality are deeply intertwined — the multi-functionality of the space is, we propose, best explained if its different uses were distributed across largely non-overlapping seasonal windows. These two phenomena are evidenced differently, a distinction we maintain throughout what follows: the multi-functional, non-simultaneous use of the spaces is directly attested by the micro- and macro-remains, whereas the placement of each use within the annual cycle is an inference from its known seasonal timing rather than a sequence read from the deposits. Future work, for example stable-isotope or sclerochronological analyses of the murex shells and faunal remains, may allow these seasonal placements to be tested directly.

The combined data indicate that the newly excavated area (the Eastern and Western Spaces; Fig 4) was utilized for four distinct purposes: olive oil extraction, purple-dye production, animal husbandry, and textile production.

The absence of multiple sedimentary laminations representing the continuous cycle of seasonal activities is an expected feature of a heavily used and regularly maintained workspace. In particular, cleaning practices and systematic earthen-based floor preparation between operational cycles would have masked the preservation of fine sedimentary laminations [83–89]. However, integrating macro- and microscopic evidence enables the identification of the specific activities undertaken at this location over the lifetime of Stratum 11. Several of these activities have a well-documented annual timing, which allows their use of the space to be placed, as an inference, within a cyclical pattern. The deposits themselves are not seasonally diagnostic; this seasonal anchoring is external to them. Olive harvesting and pressing fall in late autumn (October–December; [90,91]), and textile production follows the spring shearing season [92,93].

There are indications that the purple-dye industry was also subject to seasonal constraints. Pliny the Elder (Naturalis Historia IX, 62) states that “the most favorable season for taking these [shellfish] is after the rising of the Dog-star, or else before spring” (i.e., from late summer to late winter). While his testimony is debated [94: 26], such a seasonal restriction aligns with the logistical demands of the industry. Since thousands of mollusks are required to produce enough dye for a single vat [95], a continuous, year-round operation would be practically impossible. Consequently, the necessity of managing such a resource-intensive industry compels regulation of harvesting to align with the snails’ reproductive cycles to prevent the depletion of local populations. Despite these historical and biological indications, the seasonality of purple-dye manufacturing remains largely unexplored archaeologically, with existing assessments remaining largely inferential rather than grounded in systematic empirical investigation (e.g. [96,97]). The precise seasonality of the purple-dye industry depends on a multitude of complex factors, including fluctuations in dye quality across seasons, sea conditions affecting accessibility, and ecological population management. Resolving these variables requires a broad, dedicated study, which we plan to carry out in the future. Nevertheless, it is evident that the cycle of purple-dye production and dyeing was integrated into the periodicity of the other industries represented on the tell.

We propose that the overlapping seasonal cycles of olive pressing, purple-dye production, and textile manufacture established a multifaceted industrial schedule for the site. This framework is further substantiated by micro-geoarchaeological evidence of animal husbandry, which indicates the functional repurposing of these spaces during industrial ‘off-seasons.’ In the Eastern Space, the stratigraphic sequence of dung deposits sealed by wood ash indicates a managed transition between these phases; the ash’s alkaline properties likely sanitized the area—neutralizing odors and controlling pests [81,98]—before its return to industrial use. In the Western Space, however, the pyrotechnological evidence likely points to a different function. Since the vats themselves lack any signs of direct fire exposure (as discussed in [53]), we suggest that the fire residues found here were associated with heating water to be added to the vats, rather than heating the vats themselves. This process is often required to create the optimal thermal conditions for the dyeing solution (e.g., [95,99–101]).

The recurrence of olive presses, loom weights, and purple-stained vats within single architectural units, documented in both the current study area and in Building B283 (above), shows that multi-functional use was not a localized, *ad-hoc* adaptation. Whether this multi-functionality was organized as a synchronized, compound-wide seasonal rotation is an extrapolation beyond the sampled contexts, which we offer as the most economical reading of the patterns.

Thus, the functional flexibility observed in Shiqmona’s Stratum 11 offers a new perspective on Iron Age economy. While Iron Age archaeological research in the Southern Levant has focused extensively on defining the architectural and artifactual footprint of industrial zones, and although sediment-based and micro-archaeological analyses have been applied productively to production contexts, notably in metallurgical research, the temporal dimension of these activities has often remained in the shadow. The Mediterranean micro-regions are characterized by adaptive economic diversification through localized resource exploitation (cf. [102] for the general framework, although mainly applied to later periods). The micro-ecology of Tel Shiqmona–shaped by maritime and terrestrial resources–integrated diverse industries. On this reading, seasonality would emerge not merely as an environmental constraint but as a driver of economic optimization, requiring a ‘spatial elasticity’ in which the function of architectural units shifted in alignment with the annual cycle of production. This capacity to interweave high-prestige industries, such as purple-dye production, with the fundamental requirements of animal husbandry, olive oil extraction, and textile manufacturing, reflects a level of administrative sophistication that may also define other state-controlled economic centers of the period.

Most of the excavated Iron Age sites in the Levant are perennial settlements [e.g., 1]. However, Shiqmona’s unique character suggests there was little reason for year-round occupation unless its economic potential was being fully exploited through strategic multi-functionality. To truly comprehend this site as an ‘economic machine’ of the Kingdom of Israel, we must account for these seasonal rhythms. The questions of how seasonal cycles governed industrial scheduling, how architectural spaces were systematically repurposed between seasons, and how such dynamic multi-functionality was maintained and managed are not unique to Shiqmona; they must be integrated into the study of any activity area governed by environmental or biological windows, including olive oil and wine production, grain processing, textile manufacturing, seasonal storage, and navigation. By integrating high-resolution archaeological and micro-geoarchaeological approaches, we can now begin to ‘read’ the operational protocols of space management—from planned sanitary measures using wood ash to the systematic conversion of infrastructure between the harvest and weaving seasons.

## Conclusion

This focused analysis, integrating macro and microscopic archaeological finds with sediment analysis, reveals the dynamic complexity of spatial use, dictated by seasonal cycles and varied economic pursuits. We propose a cyclical model, consistent with the evidence, in which the studied spaces shifted between purple-dye manufacture, olive-oil pressing, and textile production. The placement of these activities within the year: olive pressing in autumn, textile production in spring; follows from their known seasonal timing rather than from the deposits, and is therefore part of the proposed model. Integral to this rotation was the sheltering of livestock during industrial ‘off-seasons,’ (probably winter), a practice that necessitated the rigorous sanitary maintenance observed in the micro-stratigraphy. While the precise seasonal window for purple dye production remains a subject for further investigation, it was clearly interwoven into this industrial rotation rather than operating as a constant, year-round activity.

Our findings support this dynamic within the studied spaces of an elite-controlled compound. However, such multi-scalar approaches are essential for comprehending any activity area governed by environmental or biological windows and economic possibilities. Ultimately, when encountering a seasonal industry (e.g., oil or wine presses), in order to truly understand the functional history of the studied space, one must ask: what happened there during the many months each year when the industry stood idle? Future research should investigate how these strategic seasonal rotations are manifested in other industrial and domestic contexts. Recognizing these seasonal rhythms is fundamental to re-evaluating the operational complexity and efficiency of the Iron Age economy.
